# NIA Coordinated Analysis of Dynamic Socioemotional Experiences and Well-Being during the Pandemic

**DOI:** 10.1093/geroni/igab046.3509

**Published:** 2021-12-17

**Authors:** Shevaun Neupert, Sumbleen Ali, Isabella Bouklas, Rita Hu, Reilly Kincaid, Niccole Nelson, Shiyang Zhang, Stacey Scott

**Affiliations:** 1 North Carolina State University, Raleigh, North Carolina, United States; 2 Stony Brook University, State University of New York, New York, United States; 3 Duke University, Durham, North Carolina, United States; 4 Social Work, Ann Arbor, Michigan, United States; 5 Purdue University, West Lafayette, Indiana, United States; 6 University of Notre Dame, University of Notre Dame, Indiana, United States; 7 The University of Texas at Austin, Austin, Texas, United States; 8 Stony Brook University, Stony Brook, New York, United States

## Abstract

We conducted a coordinated analysis of eight longitudinal studies with data collected during the COVID-19 pandemic. Our overarching aim was to examine within-person fluctuations in health and well-being during the pandemic that may differ across the adult lifespan. The studies are from different regions of the U.S. with data collected during different periods of the pandemic. These studies sampled heterogenous age groups, used diverse methods, and were harmonized on constructs. Four longitudinal studies (Notre Dame Study of Health & Well-being, Within-Family Differences Study, Social Relations Study, and the Einstein Aging Study [EAS] Covid Telephone Interviews) and four intensive, microlongitudinal studies (Daily Experience and Well-being Study, Daily COVID-19 Spring, Daily COVID-19 Fall, EAS), with data collected between March 2020 and August 2021 were analyzed. In three studies, older adults were consistently less variable (i.e., lower within-person standard deviation) in negative emotional well-being such as negative affect and depressive symptoms compared to younger adults. In four studies older adults were also less variable in stress. Evidence of better outcomes associated with social interactions was found in three studies, where within-person variability in social interactions was positively correlated with variability in positive affect across age. These findings point toward the complexities of dynamic socioemotional experiences that unfold across historical periods and across the lifespan. These within-person fluctuations could be used as a benchmark to examine long-term trajectories of well-being.

